# ^18^F‐FDG PET/CT predicts the role of neoadjuvant immunochemotherapy in the pathological response of esophageal squamous cell carcinoma

**DOI:** 10.1111/1759-7714.15024

**Published:** 2023-07-09

**Authors:** Shuohua Wang, Shouyin Di, Jing Lu, Shun Xie, Zhenyang Yu, Yingkui Liang, Taiqian Gong

**Affiliations:** ^1^ Department of Thoracic Surgery Navy Clinical College, Anhui Medical University Hefei China; ^2^ Department of Thoracic Surgery The Fifth School of Clinical Medicine, Anhui Medical University Hefei China; ^3^ Department of Thoracic Surgery The Sixth Medical Center of Chinese PLA General Hospital Beijing China; ^4^ Department of Pathology The Sixth Medical Center of Chinese PLA General Hospital Beijing China; ^5^ Department of Nuclear Medicine The Sixth Medical Center of Chinese PLA General Hospital Beijing China

**Keywords:** ^18^F‐FDG PET/CT, esophageal squamous cell carcinoma, neoadjuvant therapy, pathological response

## Abstract

**Background:**

This study aimed to investigate the predictive value of ^18^F‐FDG PET/CT for pathological response after neoadjuvant immunochemotherapy (NICT) in patients with esophageal squamous cell carcinoma (ESCC).

**Methods:**

The clinical data of 54 patients with ESCC who underwent two cycles of NICT followed by surgery were retrospectively analyzed. NICT consisted of PD‐1 blockade therapy combined with chemotherapy. ^18^F‐FDG PET/CT scans were performed before and after NICT. The pathological results after surgery were used to assess the degree of pathological response. The scan parameters of ^18^F‐FDG PET/CT and their changes before and after NICT were compared with the pathological response.

**Results:**

Among the 54 patients, 10 (18.5%) achieved complete pathological response (pCR) and 21 (38.9%) achieved major pathological response (MPR). The post‐NICT scan parameters and their changes were significantly associated with the pathological response. In addition, the values of the changes in the scanned parameters before and after treatment can further predict the pathological response of the patient.

**Conclusion:**

^18^F‐FDG PET/CT is a useful tool to evaluate the efficacy of NICT and predict pathological response in patients with ESCC. The post‐NICT scan parameters and their changes can help identify patients who are likely to achieve pCR or MPR.

## Graphical abstract

The efficacy of neoadjuvant immunochemotherapy (NICT) with PD‐1 blocked and chemotherapy was evaluated in 54 patients with esophageal squamous cell carcinnoma (ESCC) who underwent surgery after two cycles of NICT. ^18^F‐FDG PET/CT scans were used before and after NICT to measure the changes in tumor metabolism and viability, and compared with the pathological response after surgery.^18^F‐FDG PET/CT scan parameters and their changes were found to be significantly associated with the pathological response, and could help identify patients who achieved complete or major pathological response.

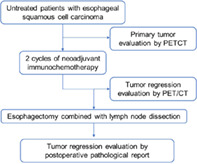

## INTRODUCTION

Previous studies have demonstrated the superiority of neoadjuvant chemoradiotherapy for the treatment of locally advanced esophageal squamous cell carcinoma (ESCC).^1^, ^2^ However, this modality is associated with high rates of adverse effects and perioperative mortality.^3^ Recently, several studies have reported promising results of neoadjuvant chemotherapy combined with programmed cell death 1 (PD‐1) blockade, which achieved significant tumor regression and low perioperative mortality.^4^, ^5^ Despite the widespread use of minimally invasive esophagectomy, postoperative mortality remains high and the patients' quality of life is significantly impaired. For patients who achieve a complete pathological response (pCR), surgery or additional neoadjuvant therapy may be unnecessary. Patients who do not achieve a major pathological response (MPR) may be resistant to neoadjuvant immunochemotherapy and require prompt surgery. The identification of patients who achieve pCR and those who do not achieve MPR after neoadjuvant immunochemotherapy remains an unresolved issue.

^18^F‐fluorodeoxyglucose (FDG) positron emission tomography/computed tomography (PET/CT) is a common technique for radiological staging of advanced ESCC^6^ as it can measure the intensity of FDG uptake and reveal the changes in tumor metabolism and viability regardless of the underlying structural changes.^7^ PET imaging technology has improved a lot in recent years, not only in imaging equipment and software, but also in imaging probes and contrast agents. Schwenck et al. reviewed the latest uses of PET imaging in cancer, such as new radioactive tracers, multimodal imaging, artificial intelligence, and personalized medicine and immunotherapy.^8^ These improvements may make PET imaging more accurate and useful in cancer diagnosis and treatment. Several studies have explored the role of PET/CT in various aspects of esophageal cancer, such as predicting the response to neoadjuvant chemotherapy,^9^ detecting postoperative tumor recurrence,^10^ and assessing other outcomes.^11^ However, the role of PET/CT in predicting the pathological response after neoadjuvant immunochemotherapy is still under‐researched. Wang et al. conducted a retrospective study and confirmed that ^18^F‐FDG PET/CT parameters can accurately predict pCR after neoadjuvant immunochemotherapy in resectable ESCC.^12^ Besides identifying patients who achieve pCR, another clinical challenge is identifying patients who do not achieve MPR. For patients who achieve pCR, surgery may be avoided; for patients who achieve MPR, another two cycles of neoadjuvant immunochemotherapy may be considered; for patients who are resistant to neoadjuvant immunochemotherapy, surgery should be performed as soon as possible. The optimal management of patients who achieve pCR is still a matter of debate, but finding a noninvasive method to assess pathological response is crucial.

This real‐world study collected data from patients who underwent neoadjuvant immunochemotherapy and esophagectomy and investigated the role of ^18^F‐FDG PET/CT parameters in assessing the pathological response of ESCC.

## METHODS

### Study design

This was a single center prospective real‐world study, approved by the Ethics Committee of the Sixth Medical Center of PLA General Hospital. The study included patients with esophageal squamous cell carcinoma who underwent neoadjuvant immunochemotherapy and esophagectomy at the Department of Thoracic Surgery, the sixth Medical Center of the Chinese PLA General Hospital, from January 2020 to December 2022. The patients underwent PET/CT examinations according to their clinical treatment assessment and provided informed consent for all treatments and examinations. We selected the pathological response after surgery as our primary endpoint, and evaluated it according to the Mandard tumor regression grade (TRG) system. All patients continued treatment and follow‐up after the experimental observation endpoint.

### Eligibility criteria

The inclusion criteria were: age between 18 and 80 years; histologically or cytologically confirmed esophageal squamous carcinoma; and no prior radiotherapy, chemotherapy, or surgery for the tumor. The exclusion criteria were: prior immunotherapy or known allergy to monoclonal or chemotherapeutic agents; history of other critical illnesses such as multiorgan failure, widespread infection, shock, stroke, or any vascular embolic event within 6 months before enrollment; severe malnutrition; or other primary malignancies. Patients who did not receive a PET/CT scan 1 week before surgery were also excluded from the analysis.

### Treatment regimen

The patients received two cycles of neoadjuvant immunochemotherapy (NICT) before surgery, tailored to their individual conditions. The NICT consisted of intravenous lobaplatin (40 mg) and paclitaxel (400 mg) on day 1, and intravenous PD‐1 monoclonal antibody (200 mg) on day 2. The PD‐1 monoclonal antibodies used were sintilimab, camrelizumab, or tislelizumab. The NICT was repeated on day 15, and surgery was performed 2 weeks after the second cycle. The surgical procedure was esophagectomy with lymph node dissection.

### PET/CT imaging

The patients underwent PET/CT imaging using a GE Discovery ST16 scanner with ^18^F‐FDG as the tracer (provided by China Atomic High Tech). The patients fasted for 6 h before the scan and had their blood glucose levels checked (4.5–6.5 mmol/L). They received an intravenous injection of ^18^F‐FDG (185–370 MBq) based on their body mass and rested for about an hour before the scan. The scan covered the region from the cranial apex to the mid‐femur, starting with a fluoroscopic acquisition, followed by a CT scan (150 mA, 120 kV), and then a 3D emission acquisition (23 min/bed, matrix 128 × 128). The CT data were used for attenuation correction and reconstructed using ordered subsets expectation maximization (OSEM). The fused images were processed with GE AW workstation software.

### PET/CT data analysis

Two experienced nuclear medicine physicians visually assessed the PET/CT images to determine the extent and morphology of the lesions and the areas of increased FDG uptake. The PET/CT scans of patients before neoadjuvant immunochemotherapy (scan 1) and before surgery (scan 2) were recorded and compared. Semi‐quantitative analysis was performed using volumetrics to measure the maximum standardized uptake value (SUV_max_), mean standardized uptake value (SUV_mean_), MTV, and TLG of the lesions at different sites. The SUV_max_ of the blood pool at the aortic arch (10 mm diameter) without including the vessel wall was measured, and the ratio of the SUV_max_ of the lesion to the SUV_max_ of the blood pool was calculated as SUV_TBR_. The changes in these parameters before and after treatment are expressed as percentages; for example, ΔSUV_max_ % = (Scan 1 SUV_max_ – Scan 2 SUV_max_)/Scan 1 SUV_max_ × 100%.

### Pathological diagnosis

The pathological response was assessed by measuring the percentage of viable tumors in the primary tumor lesions using hematoxylin–eosin (HE) staining. The specimens were independently analyzed by two senior pathologists. The patients were classified into four groups based on the tumor regression score (TRS) according to the Mandard scoring system: pCR (TRS 1, no residual tumor cells in the primary lesion and lymph nodes), non‐pCR (TRS 4–5, minimal or no tumor regression), MPR (TRS 1–2, complete or subtotal tumor regression), and non‐MPR (TRS 3–5, partial, minimal, or no tumor regression).

### Swimmer plot

We recorded the duration of treatment for each patient, and created a swimmer plot based on their imaging, pathological, and pathological response data using Microsoft Excel. A swimmer plot is a graphical tool that can display the temporal dynamics of individual patients in a clinical trial. The plot can visually show the distribution and association of imaging and pathological results among patients.

### Statistical analysis

The data were collected according to the actual condition of the patients and summarized by frequency and proportion for categorical variables. For numerical variables, descriptive statistics are reported as mean ± standard deviation or median (interquartile range), depending on whether the data followed a normal distribution, which was tested by the Shapiro–Wilk normality test. The homogeneity of variance was tested by Levene's test (based on mean). The comparison between two groups was performed by *t*‐test, Welch's *t*‐test, or Wilcoxon test depending on whether the data met the normal distribution and chi‐square test assumptions. For categorical variables, if the condition of 5 > theoretical frequency ≥1 and total sample size ≥40 was satisfied, the continuous corrected chi‐square test (Yates' correction) was used for comparison between groups. The patients were divided into pCR and non‐pCR groups, and MPR and non‐MPR groups based on postoperative pathological findings. Receiver operating characteristic (ROC) curves were used to evaluate the diagnostic performance of each metabolic parameter index. The area under the ROC curve (AUC), sensitivity, specificity, positive predictive value (PPV), negative predictive value (NPV), and accuracy with their 95% confidence intervals (95% CI) were calculated. The critical values for ROC curve analysis were calculated based on the Youden index. The differences in ROC curves were tested by DeLong's test. All data analyses were performed using R software (version 4.2.1) and the corresponding R package.

## RESULTS

### Clinical characteristics of the patients

**FIGURE 1:**
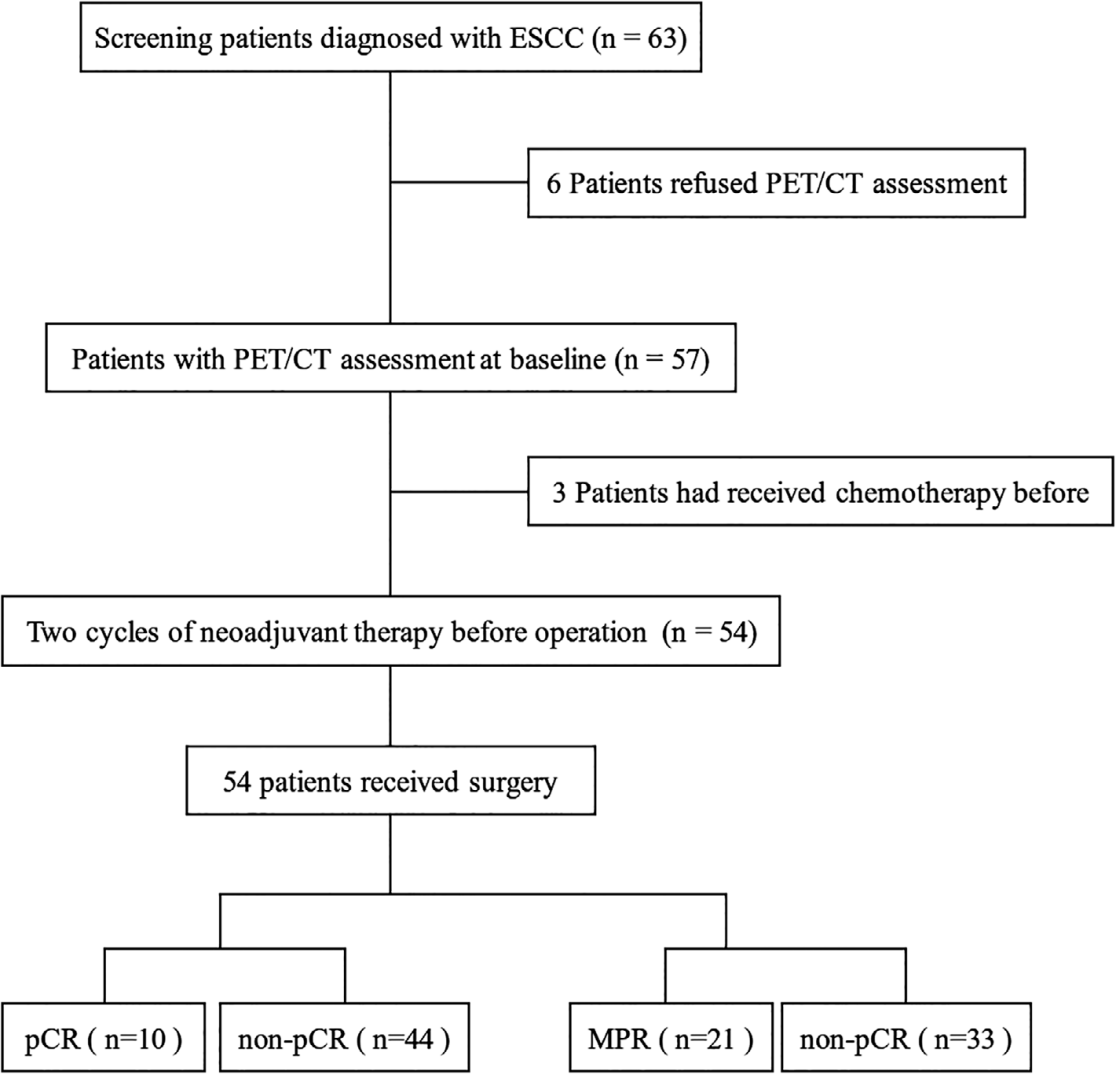
The flow chart of the study. ESCC, esophageal squamous cell carcinoma; MPR, major pathological response; pCR, pathological complete response; PET/CT, positron emission tomography/computed tomography.

This study enrolled 63 patients with esophageal squamous cell carcinoma between January 2020 and December 2022 and excluded nine patients who had prior treatment or did not undergo PET/CT before surgery. The final sample comprised 54 patients who received two cycles of neoadjuvant immunochemotherapy followed by esophagectomy (Figure 1). Tables 1 and 2 display the baseline characteristics of the patients by pathological response groups. No significant differences were observed between the groups in terms of age, gender, smoking, alcohol, tumor site, pretreatment stage, or treatment regimen. The metabolic parameters of PET/CT scans before (scan 1) and after (scan 2) neoadjuvant treatment were analyzed using descriptive and inferential statistics. Tables 3 and 4 present the results of the statistical tests for the metabolic parameters by pathological response groups. No significant differences were found in scan 1 parameters between the groups. However, in scan 2, SUV_max_, SUV_mean_, and SUV_TBR_ were significantly lower in the pCR group than in the non‐pCR group (*p* < 0.05), and also lower in the MPR group than in the non‐MPR group (*p* < 0.05). Moreover, the changes in metabolic parameters from scan 1 to scan 2 (Δmetabolic parameters) differed significantly between the groups, except for ΔMTV. ΔSUV_max_, ΔSUV_mean_, ΔSUV_TBR_, and ΔTLG were significantly lower in the pCR group than in the non‐pCR group (*p* < 0.05) and also lower in the MPR group than in the non‐MPR group (*p* < 0.05).

**TABLE 1:** Patient characteristics in the pCR and non‐pCR groups.

Characteristics	All patients (*n* = 54)	pCR (*n* = 10)	Non‐pCR (*n* = 44)	*p*‐value
Median age (range)	61 (42,76)	60 (47,76)	62 (42–76)	0.514
Gender, *n* (%)				
Male	45 (83.3%)	9 (16.7%)	36 (66.7%)	0.875
Female	9 (16.7%)	1 (1.9%)	8 (14.8%)	
Smoking, *n* (%)				
Never	21 (38.9%)	2 (3.7%)	19 (35.2%)	0.318
Current	33 (61.1%)	8 (14.8%)	25 (46.3%)	
Drinking, *n* (%)				
Never	15 (27.8%)	2 (3.7%)	13 (24.1%)	0.828
Current	39 (72.2%)	8 (14.8%)	31 (57.4%)	
Location of tumor, *n* (%)				
Cervical	1 (1.9%)	1 (1.9%)	0 (0%)	0.204
Upper thoracic	7 (12.9%)	1 (1.9%)	6 (11.1%)	
Middle thoracic	21 (38.9%)	4 (7.4%)	17 (31.5%)	
Lower thoracic	25 (46.3%)	4 (7.4%)	21 (38.9%)	
cT_stage, *n* (%)				
2	13 (24.1%)	3 (5.6%)	10 (18.5%)	0.805
3	40 (74.0%)	7 (12.9%)	33 (61.1%)	
4	1 (1.9%)	0 (0%)	1 (1.9%)	
cN_stage, *n* (%)				
0	9 (16.7%)	1 (1.9%)	8 (14.8%)	0.348
1	11 (20.3%)	4 (7.4%)	7 (12.9%)	
2	30 (55.5%)	4 (7.4%)	26 (48.1%)	
3	4 (7.5%)	1 (1.9%)	3 (5.6%)	
Clinical_stage, *n* (%)				
I	2 (3.7%)	0 (0%)	2 (3.7%)	0.880
II	11 (20.3%)	3 (5.5%)	8 (14.8%)	
III	37 (68.5%)	6 (11.1%)	31 (57.4%)	
IV	4 (7.5%)	1 (1.9%)	3 (5.6%)	
Neoadjuvant therapy regimen, *n* (%)				
Sintilimab	30 (55.6%)	5 (9.3%)	25 (46.3%)	0.189
Camrelizumab	18 (32.5%)	2 (3.7%)	15 (27.8%)	
Tislelizumab	7 (12.9%)	3 (5.6%)	4 (7.4%)	

Abbreviations: pCR, complete pathological response.

**TABLE 2:** Patient characteristics in MPR and non‐MPR groups.

Characteristics	All patients (*n* = 54)	MPR (*n* = 21)	Non‐MPR (*n* = 33)	*p*‐value
Median age (range)	61 (42,76)	63(47,76)	60(42–76)	0.231
Gender, *n* (%)				
Male	45 (83.3%)	17 (31.5%)	28 (51.9%)	1.000
Female	9 (16.7%)	4 (7.4%)	5 (9.3%)	
Smoking, *n* (%)				
Never	21 (38.9%)	8 (14.8%)	13 (24.1%)	0.924
Current	33 (61.1%)	13 (24.1%)	20 (37%)	
Drinking, *n* (%)				
Never	15 (27.8%)	8 (14.8%)	7 (13%)	0.177
Current	39 (72.2%)	13 (24.1%)	26 (48.1%)	
Location of tumor, *n* (%)				
Cervical	1 (1.9%)	1 (1.9%)	0 (0%)	0.593
Upper thoracic	7 (12.9%)	2 (3.7%)	5 (9.3%)	
Middle thoracic	21 (38.9%)	8 (14.8%)	13 (24.1%)	
Lower thoracic	25 (46.3%)	10 (18.5%)	15 (27.8%)	
cT_stage, *n* (%)				
2	13 (24.1%)	7 (13%)	6 (11.1%)	0.404
3	40 (74.0%)	14 (25.9%)	26 (48.1%)	
4	1 (1.9%)	0 (0%)	1 (1.9%)	
cN_stage, *n* (%)				
0	9 (16.7%)	1 (1.9%)	8 (14.8%)	0.097
1	11 (20.3%)	6 (11.1%)	5 (9.3%)	
2	30 (55.5%)	11 (20.4%)	19 (35.2%)	
3	4 (7.5%)	3 (5.6%)	1 (1.9%)	
Clinical_stage, *n* (%)				
I	2 (3.7%)	0 (0%)	2 (3.7%)	0.247
II	11 (20.3%)	3 (5.6%)	8 (14.8%)	
III	37 (68.5%)	15 (27.8%)	22 (40.7%)	
IV	4 (7.5%)	3 (5.6%)	1 (1.9%)	
Neoadjuvant therapy regimen, *n* (%)				
Sintilimab	30 (55.6%)	13 (24.1%)	17 (31.5%)	0.232
Camrelizumab	18 (32.5%)	4 (7.4%)	13 (24.1%)	
Tislelizumab	7 (12.9%)	4 (7.4%)	3 (5.6%)	

Abbreviations: MPR, major pathological response.

**TABLE 3:** The characteristics of PET/CT parameters of complete pathological response.

Characteristics	pCR	Non‐pCR	*p* value
Scan 1^a^			
SUV_max_	17.007 ± 2.222	14.739 ± 5.888	0.141
SUV_mean_	11.684 ± 2.1863	9.595 ± 4.0425	0.235
SUV_TBR_	9.940 ± 1.9524	7.448 ± 3.248	0.084
MTV	13.692 (10.464, 15.819)	10.464 (6.748, 12.42)	0.355
TLG	141.33 (110.19, 206.5)	106.29 (42.383, 151.3)	0.227
Scan 2^b^			
SUV_max_	2.6955 (2.268, 3.033)	7.571 (3.473, 13.809)	**<0.001**
SUV_mean_	1.582 (1.382, 1.796)	4.121 (2.28, 8.390)	**<0.001**
SUV_TBR_	1.3159 (1.19, 1.57)	3.916 (1.697, 6.528)	**<0.001**
MTV	6.699 (2.641, 17.586)	4.6945 (3.0315, 7.8727)	0.300
TLG	18.221 (6.815, 25.011)	20.51 (9.464, 48.763)	0.561
The changes between scan 1 and scan 2^a^ (Δ%)			
ΔSUV_max_	83.57 (79.928, 86.737)	45.067 (1.175, 82.503)	**<0.01**
ΔSUV_mean_	86.314 ± 3.7523	39.154 ± 40.99	**<0.001**
ΔSUV_TBR_	85.443 ± 4.1584	35.845 ± 42.101	**<0.001**
ΔMTV	3.762 (−15.228, 32.465)	35.13 (13.582, 63.293)	0.190
ΔTLG	82.604 (70, 88.863)	70.193 (42.251, 86.095)	0.291

*Note*: Values in bold are *p* < 0.05.

*Note*: Mean ± SD is used for those with normal distribution, and median (IQR) is used for those who do not conform to normal distribution. MTV, metabolic tumor volume; SUV_max_, maximum standardized uptake value. SUV_mean_, mean standardized uptake value. SUV_TBR_, the tumor‐to‐blood pool SUV_max_ ratio; TLG, total lesion glycolysis. n^a^ were patients who underwent PET/CT scan before and after neoadjuvant therapy, including six pCR patiernts and 25 non‐pCR patients. n^b^ were patients who underwent PET/CT scan before operation, including 10 pCR patiernts and 44 non‐pCR patients.

**TABLE 4:** The characteristics of PET/CT parameters of major pathological response.

Characteristics	MPR	Non‐MPR	*p* value
Scan 1^a^			
SUV_max_	17.322 ± 3.762	13.825 ± 5.945	0.080
SUV_mean_	11.623 ± 2.820	8.9745 ± 4.070	0.058
SUV_TBR_	8.834 ± 2.660	7.360 ± 3.403	0.214
MTV	13.692 (8.360, 17.163)	9.584 (6.797, 12.127)	0.330
TLG	162.76 ± 112.92	99.115 ± 68.065	0.059
Scan 2^b^			
SUV_max_	3.066 (2.325, 4.411)	8.826 (4.287, 14.176)	**0.002**
SUV_mean_	1.829 (1.633, 2.683)	4.321 (2.332, 8.529)	**0.006**
SUV_TBR_	1.560 (1.264, 2.105)	4.687 (1.998, 6.466)	**0.002**
MTV	3.814 (2.249, 10.562)	5.574 (3.227, 9.291)	0.664
TLG	11.427 (3.536, 31.681)	20.853 (10.714, 55.59)	0.062
The changes between scan 1 and scan 2^a^ (Δ%)			
ΔSUV_max_	82.093 (55.412, 85.01)	37.692 (−2.987, 74.838)	**0.028**
ΔSUV_mean_	81.785 (56.080, 86.521)	41.805 (3.167, 77.705)	**0.039**
ΔSUV_TBR_	80.312 (57.183, 86.052)	44.46 (−4.676, 68.083)	**0.043**
ΔMTV	28.497 ± 47.727	24.192 ± 41.669	0.793
ΔTLG	84.763 (69.821, 89.879)	61.912 (36.457, 82.915)	**0.020**

*Note*: Values in bold are *p* < 0.05.

*Note*: Mean ± SD is used for those with normal distribution, and median (IQR) is used for those who do not conform to normal distribution. MTV, metabolic tumor volume; PET/CT, positron emission tomography/computed tomography; SUV_max_, maximum standardized uptake value; SUV_mean_, mean standardized uptake value; SUV_TBR_, the tumor‐to‐blood pool SUV_max_ ratio; TLG, total lesion glycolysis. n^a^ were patients who underwent PET/CT scan before and after neoadjuvant therapy, including 12 MPR patients and 19 non‐MPR patients. n^b^ were patients who underwent PET/CT scan before operation, including 21 MPR patients and 33 non‐MPR patients.

### Treatment and analysis of the subjects

**FIGURE 2:**
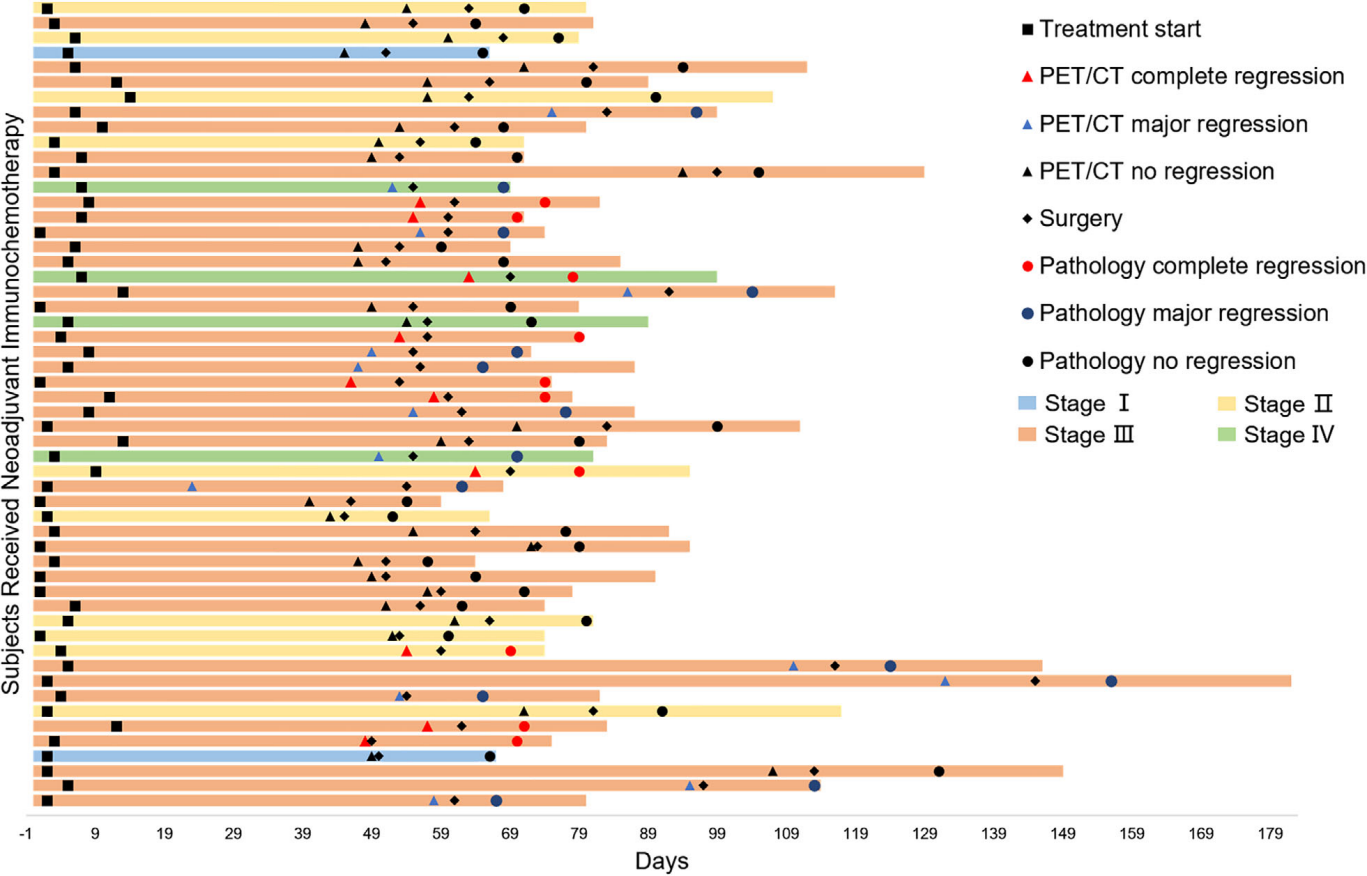
The swimmer plot of the treatment status of the subjects. Each horizontal bar represents the time from admission to discharge after operation, and different colors indicate the clinical stage of the tumor at the time of treatment; squares represent the start time of the first cycle of neoadjuvant immunotherapy for the patient; triangles represent the tumor regression status calculated based on positron emission tomography/computed tomography (PET/CT) results after two complete treatment cycles, where red represents complete tumor regression, blue represents major tumor regression, and black represents no tumor regression; diamonds represent the time when the patient underwent surgery; circles represent the tumor regression status based on pathological analysis, where red represents complete tumor regression, blue represents major tumor regression, and black represents no tumor regression; all patients continued treatment and follow‐up after the experimental observation endpoint.

In addition, we created a swimmer plot based on the treatment status of 54 patients and found that the tumor regression status measured by PET/CT examination was consistent with the tumor regression status reported by pathological analysis (Figure 2). The swimmer plot shows the time span from each patient's first admission and treatment to discharge after surgery, as well as the start time of the first cycle of neoadjuvant immunotherapy, the tumor regression status after two complete treatment cycles, the time of surgery, and the tumor regression status based on pathological analysis. The tumor regression status is indicated by different colors and shapes: red for complete tumor regression, blue for major tumor regression, and black for no tumor regression.

### PET/CT parameters were significantly different in different pathology subgroups

The metabolic parameters of PET/CT scans before (scan 1) and after (scan 2) neoadjuvant treatment were compared between different pathological response groups using *t*‐tests. Tables 3 and 4 show the means, standard deviations, and *p*‐values of the metabolic parameters by group. No significant differences in scan 1 parameters were found between the groups (*p* > 0.05). However, in scan 2, the pCR group had significantly lower SUV_max_, SUV_mean_, and SUV_TBR_ than the non‐pCR group (*p* < 0.05), and the MPR group had significantly lower SUV_max_, SUV_mean_, and SUV_TBR_ than the non‐MPR group (*p* < 0.05). The changes in metabolic parameters from scan 1 to scan 2 (Δmetabolic parameters) were also calculated and showed that ΔSUV_max_, ΔSUV_mean_, ΔSUV_TBR_, and ΔTLG were significantly lower in the pCR group than in the non‐pCR group (*p* < 0.05), and also lower in the MPR group than in the non‐MPR group (*p* < 0.05). The only exception was ΔMTV, which did not differ significantly between the groups (*p* > 0.05).

### Predictive value of metabolic parameters for complete disease remission

**FIGURE 3:**
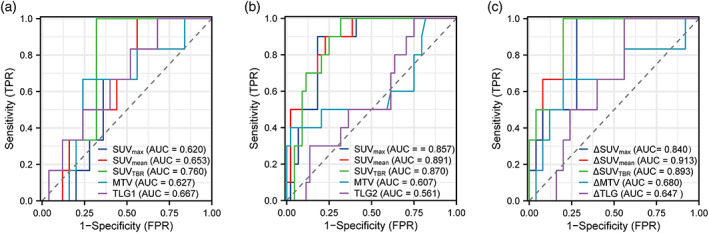
Receiver operating characteristic (ROC) curve for metabolic parameters against the complete pathological response. AUC, area under the curve. The data based on (a) scan 1a, (b) scan 2b and (c) the changes between scan 1 and scan 2a.

To examine the predictive value of metabolic parameters for pCR, ROC analysis was performed using scan 1 and scan 2 parameters (Table 5, Figure 3a–c). In scan 1, only SUV_TBR_ (AUC = 0.760) showed a significant ability to discriminate between pCR and non‐pCR patients (Figure 3a). In scan 2, SUV_max_ (AUC = 0.857), SUV_mean_ (AUC = 0.891), and SUV_TBR_ (AUC = 0.870) were all significant predictors of pCR. Among them, SUV_mean_ had the highest AUC value (AUC = 0.891, 95% CI: 0.795–0.987) with a cutoff value of 2.188, resulting in a PPV of (9/54) 47.3% and a NPV of (34/54) 97.1% (Table 3, Figure 3b). No significant differences were found between the ROC curves of these parameters using DeLong's test. The changes in metabolic parameters from scan 1 to scan 2 (Δmetabolic parameters) were also analyzed for their predictive value for pCR (Figure 3c). ΔSUV_max_ (AUC = 0.840), ΔSUV_mean_ (AUC = 0.913), and ΔSUV_TBR_ (AUC = 0.893) were all significant predictors of pCR.

**TABLE 5:** Different metabolic parameters on predicting tumor pathological complete response.

	AUC (95% CI)	Cut‐off	Sensitivity%	Specificity%	Accuracy%	PPV%	NPV%	Youden index
Scan 1^a^								
SUV_max_	0.620 (0.423–0.817)	13.62	100.0	44.0	54.8	30.0	100.0	0.44
SUV_mean_	0.653 (0.440–0.867)	8.55	100.0	44.0	54.8	30.0	100.0	0.44
SUV_TBR_	0.760 (0.585–0.935)	8.2778	100.0	68.0	74.2	42.9	100.0	0.68
MTV	0.627 (0.373–0.880)	13.056	66.7	76.0	74.2	40.0	90.5	0.43
TLG	0.667 (0.435–0.898)	44.219	100.0	32.0	45.2	26.1	100.0	0.32
Scan 2^b^								
SUV_max_	0.857 (0.752–0.962)	3.1965	90.0	81.8	83.3	52.9	97.3	0.72
SUV_mean_	0.891 (0.795–0.987)	2.188	90.0	77.3	79.6	47.4	97.1	0.67
SUV_TBR_	0.870 (0.774–0.967)	2.0936	100.0	68.2	74.1	41.7	100.0	0.68
MTV	0.607 (0.365–0.848)	13.607	40.0	97.7	87.0	80.0	87.8	0.38
TLG	0.561 (0.375–0.748)	52.094	100.0	25.0	38.9	23.3	100.0	0.25
The changes between scan 1 and scan 2a (Δ%)								
ΔSUV_max_	0.840 (0.689–0.991)	74.826	100.0	72.0	77.4	46.2	100.0	0.72
ΔSUV_mean_	0.913 (0.809–1.000)	81.568	100.0	80.0	83.9	54.5	100.0	0.80
ΔSUV_TBR_	0.893 (0.774–1.000)	79.931	100.0	80.0	83.9	54.5	100.0	0.80
ΔMTV	0.680 (0.386–0.974)	11.656	66.7	80.0	77.4	44.4	90.9	0.47
ΔTLG	0.647 (0.440–0.854)	65.57	100.0	44.0	54.8	30.0	100.0	0.44

Abbreviations: AUC, area under the ROC curve; NPV, negative predictive value; PPV, positive predictive value; SUV_max_, maximum standardized uptake value; SUV_mean_, mean standardized uptake value; SUV_TBR_, the tumor‐to‐blood pool SUV_max_ ratio; TLG, total lesion glycolysis.

### Predictive value of metabolic parameters for disease remission

**FIGURE 4:**
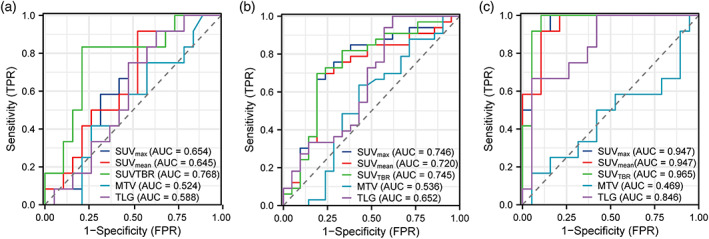
Receiver operating characteristic (ROC) curve for metabolic parameters against the major pathological response. AUC, area under the curve. The data based on (a) scan 1a, (b) scan 2b and (c) the changes between scan 1 and scan 2a.

**FIGURE 5:**
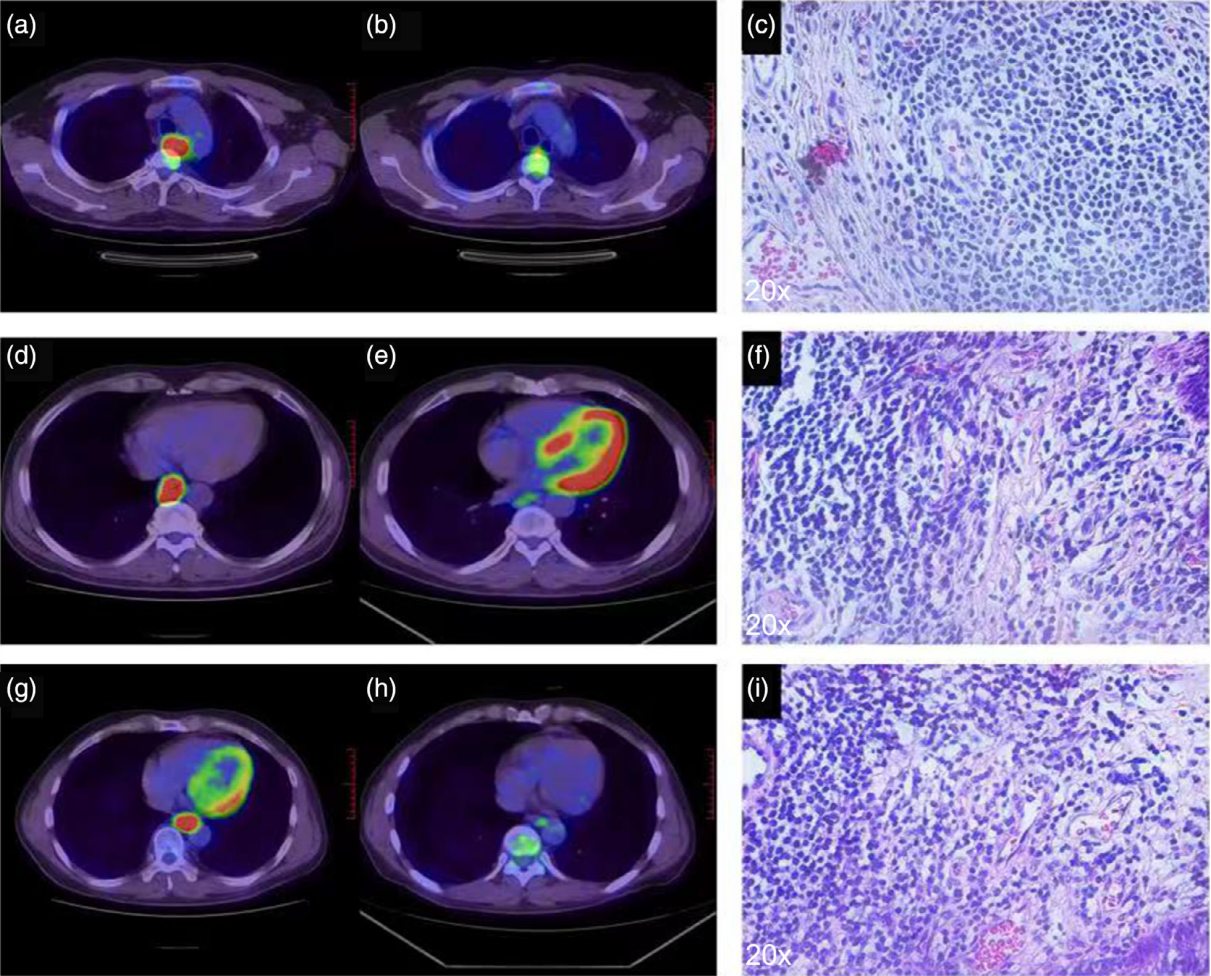
Positron emission tomography/computed tomography (PET/CT) and immunohistochemical results of typical patients. Typical patient ^18^F‐ fluorodeoxyglucose (FDG) PET/CT and pathological immunohistochemistry. (a, b and c) A typical pCR patient; (a) before neoadjuvant therapy, (b) after two cycles of neoadjuvant therapy and (c) the pathological specimen after operation. (d, e and f) A typical major pathological response (MPR) patient. (g, h and i) A typical non‐MPR patient.

The ROC analysis for identifying MPR yielded similar results to those for identifying pCR (Table 6, Figure 5d–f). In scan 1, only SUV_TBR_ (AUC = 0.768) was a significant predictor of MPR (Figure 4a). In scan 2, SUV_max_ (AUC = 0.746), SUV_mean_ (AUC = 0.720), and SUV_TBR_ (AUC = 0.745) were all significant predictors of MPR (Figure 4b). Among the Δmetabolic parameters, ΔSUV_max_ (AUC = 0.947), ΔSUV_mean_ (AUC = 0.947), ΔSUV_TBR_ (AUC = 0.965), and ΔTLG (AUC = 0.846) were all significant predictors of MPR (Figure 4c). ΔSUV_TBR_ had the highest AUC value (AUC = 0.965, 95% CI: 0.795–0.987) with a cutoff value of 0.572, resulting in a PPV of (12/31) 85.7% and a NPV of (17/31) 100%. The ROC curves of these parameters were significantly different from each other using DeLong's test. Figure 5g–i shows typical images and pathological examinations of patients with unimproved pathological findings after treatment.

**TABLE 6:** Different metabolic parameters on predicting tumor major pathological response.

	AUC (95% CI)	Cutoff	Sensitivity%	Specificity%	Accuracy%	PPV%	NPV%	Youden index
Scan 1a								
SUV_max_	0.654 (0.457, 0.850)	12.747	91.7	47.4	64.5	52.4	90.0	0.39
SUV_mean_	0.645 (0.445, 0.844)	7.8035	91.7	47.4	64.5	52.4	90.0	0.39
SUV_TBR_	0.768 (0.587, 0.948)	8.2778	83.3	78.9	80.6	71.4	88.2	0.62
MTV	0.524 (0.312, 0.736)	9.2405	75.0	42.1	54.8	45.0	72.7	0.17
TLG	0.588 (0.384, 0.792)	44.219	91.7	36.8	58.1	47.8	87.5	0.29
Scan 2b								
SUV_max_	0.746 (0.600, 0.892)	4.4405	72.7	76.2	74.1	82.8	64.0	0.49
SUV_mean_	0.720 (0.571, 0.869)	2.8225	69.7	80.9	74.1	85.2	62.9	0.51
SUV_TBR_	0.745 (0.598, 0.891)	2.4833	69.7	80.9	74.1	85.2	62.9	0.51
MTV	0.536 (0.363, 0.709)	4.254	63.6	57.1	61.1	70.0	50.0	0.21
TLG	0.652 (0.490, 0.814)	5.1635	100.0	38.1	75.9	71.7	100.0	0.38
The changes between scan 1 and scan 2a (Δ%)								
ΔSUV_max_	0.947 (0.874, 1.000)	0.55529	100.0	84.2	90.3	80.0	100.0	0.84
ΔSUV_mean_	0.947 (0.876, 1.000)	0.76092	91.7	89.5	90.3	84.6	94.4	0.81
ΔSUV_TBR_	0.965 (0.902, 1.000)	0.57176	100.0	89.5	93.5	85.7	100.0	0.89
ΔMTV	0.469 (0.242, 0.696)	0.73529	16.7	94.7	64.5	66.7	64.3	0.11
ΔTLG	0.846 (0.706, 0.987)	0.86833	66.7	94.7	83.9	88.9	81.8	0.61

Abbreviations: AUC, area under the ROC curve; NPV, negative predictive value; PPV, positive predictive value; SUV_max_, maximum standardized uptake value; SUV_mean_, mean standardized uptake value; SUV_TBR_, the tumor‐to‐blood pool SUV_max_ ratio; TLG, total lesion glycolysis.

## DISCUSSION

This study investigated the prognostic significance of ^18^F‐FDG PET/CT for the pathological response of ESCC following neoadjuvant immunochemotherapy. In contrast to Wang et al., this study employed multiple PD‐1 inhibitors, which enhanced its validity. The study retrospectively analyzed 54 patients and examined the association between ^18^F‐FDG PET/CT and pathological response, with the aim of identifying patients with pCR, MPR, and non‐MPR after neoadjuvant immunochemotherapy. The results indicated that SUV_max_, SUV_mean_ in scan 2 and ΔSUV_max_, ΔSUV_mean_, ΔSUV_TBR_ had high predictive accuracy for the pCR and MPR of ESCC.

This study showed that SUV_max_ (AUC 0.857, cutoff value 3.20), SUV_mean_ (AUC 0.891, cutoff value 2.188) in scan 2, and ΔSUV_max_ (AUC 0.84, cutoff value 74.826), ΔSUV_mean_ (AUC 0.913, cutoff value 81.568), and ΔSUV_TBR_ (AUC 0.893, cutoff value 79.931) had a significant association with pCR with sensitivity of 90%, 90%,100%, 100%, 100%, specificity of 81.8%, 77.3%, 72.0%, 80.0%, 80.0%, and PPV of 52.9%, 47.4%,46.2%, 54.5% 54.5%, respectively. These results confirmed that ^18^F‐FDG PET/CT is a reliable method to evaluate pathological response after NICT. The role of SUV_max_ was inconsistent in previous studies. Some studies reported that SUV_max_ of ^18^F‐FDG PET/CT was not a strong predictor of pathological response of ESCC after NCRT due to esophagitis.^13^ However, Sasaki et al. found a correlation between pathological response and SUV_max_ after nCRT.^14^ Wang et al. concluded that SUV_max_ and SUV_mean_ were adequate predictors of pCR after NICT (AUC 0.848),^12^ which was in agreement with the present study (AUC 0.857). Regarding SUV_mean_, Hu et al. found that SUV_mean_ was significantly higher in nonresponders than in responders. Receiver operating characteristics curve analysis identified SUV_mean_ (AUC = 0.870, *p* = 0.010) as significant predictors of the response to concurrent chemoradiotherapy.^11^ Volume‐based parameter SUV_TBR_ might have better predictive performance in ESCC after NICT. To the best of our knowledge, only the study by Wang et al. investigated SUV_TBR_ in ESCC. In their study, SUV_TBR_ (AUC 0.860) was better than ΔSUV_TBR_ (AUC 0.668). However, our study showed that ΔSUV_TBR_ (AUC 0.893) was better than SUV_TBR_ (AUC 0.870) because of higher specificity (80% vs. 68.2%). The above differences between the study by Wang et al. and our study might be due to three aspects.^12^ First, our study was retrospective and the study by Wang et al. was prospective. Second, we enrolled patients receiving sarilumab, tirelizumab and camrelizumab, while only camrelizumab was used in the study by Wang et al.

NICT has brought remarkable changes to ESCC treatment for its impressive pCR and MPR rate and tolerable adverse effect rate.^15^, ^16^, ^17^ For patients with MPR after two cycles of NICT, pCR may be achieved after another two cycles of NICT. Distinguishing MPR from non‐MPR is another clinical challenge. To the best of our knowledge, this is the first study to report the role of ^18^F‐FDG PET/CT in predicting the major pathological response to NICT of resectable ESCC. In this study, we further evaluated the predictive performance of ^18^F‐FDG PET/CT in ESCC patients. ΔSUV_max_ (AUC 0.947, cutoff value 0.56), ΔSUV_mean_ (AUC 0.947, cutoff value 0.76) and ΔSUV_TBR_ (AUC 0.965, cutoff value 0.57) were associated with MPR with sensitivity of 100%, 91.7%, 100%, specificity of 84.2%, 89.5%, 89.5% and PPV of 80.0%, 84.6%, 85.7%, respectively. Unlike the first half of this study, metabolic parameters of scan 2 had poor predictive performance in assessing MPR.

Previous studies have examined the association between PET/CT and major pathological response.^18^, ^19^ However, the reported accuracy to predict major pathological response differs significantly among studies. In most studies, the SUV_max_ on pre‐ or post‐treatment ^18^F‐FDG PET/CT, as well as a (relative) change in SUV_max_, were analyzed. However, SUV_max_ or reduction of SUV_max_ had poor predictive performance in ESCC patients.^20^, ^21^, ^22^ Simoni et al. found that MTV and TLG before IC (scan 1), and SUV_mean_ and TLG after IC (scan 2) were related to MPR in ESCC patients who received NCRT.^23^ The study by Stiekema et al. showed that SUV_max_, MTV, and TLG played a role in identifying MPR, but the AUC of ROC in predicting MPR was 0.70 (95% CI: 0.65–0.92) for ΔSUV_max_, and 0.73 (95% CI: 0.58–0.88) for both MTV and TLG.^24^ The study by Stiekema et al. concluded that the accuracy for predicting a complete or major pathological response was limited.^24^ The conflicting results might be due to various factors, such as small sample size, esophagitis after NCRT and systemic errors. The study by Wang et al. focused on pCR and lymph nodes.^12^ Few studies have investigated the MPR predictive performance of ^18^F‐FDG PET/CT in ESCC patients who received NICT.

Identifying pCR and MPR is a clinical challenge. For patients with pCR, active surveillance may be sufficient. For patients with MPR, they may achieve pCR after another 1–2 cycles of NICT. Whether surgery is necessary for patients with pCR should be confirmed by further randomized controlled trials. However, it is crucial to find an accurate assessment of residual disease after NICT. A previous study and our study demonstrated the excellent performance of ^18^F‐FDG PET/CT for predicting pCR in resectable ESCC after NICT.^12^ Moreover, ^18^F‐FDG PET/CT is also a good predictor of MPR as shown in this study. Future clinical trials are needed to determine SUV threshold and cutoff value for pCR and MPR assessment.

Furthermore, we noticed that our analysis results were not entirely consistent with some previously published articles.^20^, ^25^, ^26^ We concluded that was related to the selection of the study population and the treatment regimen. We only included Chinese patients with ESCC in our study and excluded adenocarcinoma patients. In addition, we inferred that radiotherapy‐induced esophagitis and inflammation may cause false‐positive PET/CT results and statistical errors.^27^, ^28^, ^29^ There are some limitations in this study. First, it was a single‐center retrospective study with a small sample size. Second, we enrolled patients who received multiple PD‐1 inhibitors, including sarilumab, tirelizumab and camrelizumab. A univariate randomized controlled study would make the conclusions more convincing. Third, predicting lymphatic metastasis is important for ESCC staging. We will evaluate the predictive performance of ^18^F‐FDG PET/CT for lymphatic metastasis in future research.

In summary, this study showed that ^18^F‐FDG PET/CT is an excellent tool to predict pCR and MPR after NICT in patients with locally advanced ESCC. Future studies are still required to investigate the detailed SUV threshold and cutoff value of ^18^F‐FDG PET/CT. Additionally, studies with longer follow‐up time will elucidate the role of ^18^F‐FDG PET/CT in predicting the overall survival of ESCC after NICT. We also expect that more high‐quality and high‐sensitivity PET imaging probes and methods will provide more accurate and comprehensive diagnosis and evaluation for ESCC patients in the future.

## AUTHOR CONTRIBUTIONS

SW, SD and JL: designed the study, analyzed experimental data and drafted the manuscript. SX: participated in the experiment and manuscript preparation. ZY: analyzed experimental data. TG and YL: conceived the study, evaluated the data, and prepared the manuscript. All authors contributed to the article and approved the submitted version.

## FUNDING INFORMATION

This work was supported by Innovation Cultivation Fund of the Sixth Medical Center of PLA General Hospital (no. CXPY202003).

## CONFLICT OF INTEREST STATEMENT

The authors declare that the research was conducted in the absence of any commercial or financial relationships that could be construed as a potential conflict of interest.

## Data Availability

All relevant data generated or analyzed during this study are included in the published article.

## References

[1] Shapiro J , van Lanschot JJB , Hulshof M , et al. Neoadjuvant chemoradiotherapy plus surgery versus surgery alone for oesophageal or junctional cancer (CROSS): long‐term results of a randomised controlled trial. Lancet Oncol. 2015;16:1090–8. 26254683 10.1016/S1470-2045(15)00040-6

[2] Yang H , Liu H , Chen Y , Zhu C , Fang W , Yu Z , et al. Neoadjuvant chemoradiotherapy followed by surgery versus surgery alone for locally advanced squamous cell carcinoma of the esophagus (NEOCRTEC5010): a phase III multicenter, randomized. Open‐Label Clinical Trial J Clin Oncol. 2018;36:2796–803. 30089078 10.1200/JCO.2018.79.1483 PMC6145832

[3] Furukawa T , Hamai Y , Hihara J , et al. Impact of interval between neoadjuvant chemoradiation and surgery upon morbidity and survival of patients with squamous cell carcinoma of thoracic esophagus. Anticancer Res. 2018;38:5239–45. 30194173 10.21873/anticanres.12848

[4] Xia P , Li P , Wu S , Wang Y , Ye P , Zhang C , et al. Evaluation of the safety and effectiveness of neoadjuvant combined chemoimmunotherapy in the treatment of locally advanced esophageal squamous cell carcinoma: a retrospective single‐arm cohort study. Ann Transl Med. 2022;10:991. 36267734 10.21037/atm-22-4268 PMC9577786

[5] Zhang Z , Ye J , Li H , Gu D , du M , Ai D , et al. Neoadjuvant sintilimab and chemotherapy in patients with resectable esophageal squamous cell carcinoma: a prospective, single‐arm, phase 2 trial. Front Immunol. 2022;13:1031171. 36311804 10.3389/fimmu.2022.1031171 PMC9606329

[6] Lim CH , Park YJ , Shin M , Cho YS , Choi JY , Lee KH , et al. Tumor SUVs on 18F‐FDG PET/CT and aggressive pathological features in esophageal squamous cell carcinoma. Clin Nucl Med. 2020;45:e128–e33. 31977480 10.1097/RLU.0000000000002926

[7] Yap WK , Chang YC , Hsieh CH , Chao YK , Chen CC , Shih MC , et al. Favorable versus unfavorable prognostic groups by post‐chemoradiation FDG‐PET imaging in node‐positive esophageal squamous cell carcinoma patients treated with definitive chemoradiotherapy. Eur J Nucl Med Mol Imaging. 2018;45:689–98. 29188300 10.1007/s00259-017-3901-3

[8] Schwenck J , Sonanini D , Cotton JM , Rammensee HG , la Fougère C , Zender L , et al. Advances in PET imaging of cancer. Nat Rev Cancer. 2023;23(7):474–90. 10.1038/s41568-023-00576-4 37258875

[9] Martinez A , Infante JR , Quiros J , et al. Baseline (18)F‐FDG PET/CT quantitative parameters as prognostic factors in esophageal squamous cell cancer. Rev Esp Med Nucl Imagen Mol (Engl ed). 2022;41:164–70. 34452867 10.1016/j.remnie.2021.07.006

[10] Kaida H , Yasuda T , Shiraishi O , Kato H , Kimura Y , Hanaoka K , et al. The usefulness of the total metabolic tumor volume for predicting the postoperative recurrence of thoracic esophageal squamous cell carcinoma. BMC Cancer. 2022;22:1176. 36376801 10.1186/s12885-022-10281-4 PMC9664655

[11] Hu X , Zhou T , Ren J , Duan J , Wu H , Liu X , et al. Response prediction using (18)F‐FAPI‐04 PET/CT in patients with esophageal squamous cell carcinoma treated with concurrent chemoradiotherapy. J Nucl Med. 2022;64:625–31. 36229183 10.2967/jnumed.122.264638

[12] Wang X , Yang W , Zhou Q , Luo H , Chen W , Yeung SCJ , et al. The role of (18)F‐FDG PET/CT in predicting the pathological response to neoadjuvant PD‐1 blockade in combination with chemotherapy for resectable esophageal squamous cell carcinoma. Eur J Nucl Med Mol Imaging. 2022;49:4241–51. 35732974 10.1007/s00259-022-05872-z

[13] de Gouw D , Klarenbeek BR , Driessen M , et al. Detecting pathological complete response in esophageal cancer after neoadjuvant therapy based on imaging techniques: a diagnostic systematic review and meta‐analysis. J Thorac Oncol. 2019;14:1156–71. 30999111 10.1016/j.jtho.2019.04.004

[14] Sasaki K , Uchikado Y , Okumura H , et al. Role of (18)F‐FDG‐PET/CT in esophageal squamous cell carcinoma after neoadjuvant chemoradiotherapy. Anticancer Res. 2017;37:859–64. 28179343 10.21873/anticanres.11390

[15] Jing SW , Zhai C , Zhang W , He M , Liu QY , Yao JF , et al. Comparison of neoadjuvant immunotherapy plus chemotherapy versus chemotherapy alone for patients with locally advanced esophageal squamous cell carcinoma: a propensity score matching. Front Immunol. 2022;13:970534. 36275724 10.3389/fimmu.2022.970534 PMC9585292

[16] He W , Leng X , Mao T , Luo X , Zhou L , Yan J , et al. Toripalimab plus paclitaxel and carboplatin as neoadjuvant therapy in locally advanced Resectable esophageal squamous cell carcinoma. Oncologist. 2022;27:e18–28. 35305102 10.1093/oncolo/oyab011 PMC8842349

[17] Waters JK , Reznik SI . Update on Management of Squamous Cell Esophageal Cancer. Curr Oncol Rep. 2022;24:375–85. 35142974 10.1007/s11912-021-01153-4

[18] Ott K , Weber W , Siewert JR . The importance of PET in the diagnosis and response evaluation of esophageal cancer. Dis Esophagus. 2006;19:433–42. 17069585 10.1111/j.1442-2050.2006.00617.x

[19] Schneider PM , Eshmuminov D , Rordorf T , Vetter D , Veit‐Haibach P , Weber A , et al. (18)FDG‐PET‐CT identifies histopathological non‐responders after neoadjuvant chemotherapy in locally advanced gastric and cardia cancer: cohort study. BMC Cancer. 2018;18:548. 29743108 10.1186/s12885-018-4477-4 PMC5944162

[20] Piessen G , Petyt G , Duhamel A , Mirabel X , Huglo D , Mariette C . Ineffectiveness of (1)(8)F‐fluorodeoxyglucose positron emission tomography in the evaluation of tumor response after completion of neoadjuvant chemoradiation in esophageal cancer. Ann Surg. 2013;258:66–76. 23470576 10.1097/SLA.0b013e31828676c4

[21] Cerfolio RJ , Bryant AS , Ohja B , Bartolucci AA , Eloubeidi MA . The accuracy of endoscopic ultrasonography with fine‐needle aspiration, integrated positron emission tomography with computed tomography, and computed tomography in restaging patients with esophageal cancer after neoadjuvant chemoradiotherapy. J Thorac Cardiovasc Surg. 2005;129:1232–41. 15942562 10.1016/j.jtcvs.2004.12.042

[22] Levine EA , Farmer MR , Clark P , Mishra G , Ho C , Geisinger KR , et al. Predictive value of 18‐fluoro‐deoxy‐glucose‐positron emission tomography (18F‐FDG‐PET) in the identification of responders to chemoradiation therapy for the treatment of locally advanced esophageal cancer. Ann Surg. 2006;243:472–8. 16552197 10.1097/01.sla.0000208430.07050.61 PMC1448953

[23] Simoni N , Rossi G , Benetti G , Zuffante M , Micera R , Pavarana M , et al. (18)F‐FDG PET/CT metrics are correlated to the pathological response in esophageal cancer patients treated with induction chemotherapy followed by neoadjuvant chemo‐radiotherapy. Front Oncol. 2020;10:599907. 33330097 10.3389/fonc.2020.599907 PMC7729075

[24] Stiekema J , Vermeulen D , Vegt E , Voncken FEM , Aleman BMP , Sanders J , et al. Detecting interval metastases and response assessment using 18F‐FDG PET/CT after neoadjuvant chemoradiotherapy for esophageal cancer. Clin Nucl Med. 2014;39:862–7. 25140549 10.1097/RLU.0000000000000517

[25] Kukar M , Alnaji RM , Jabi F , Platz TA , Attwood K , Nava H , et al. Role of repeat 18F‐fluorodeoxyglucose positron emission tomography examination in predicting pathologic response following neoadjuvant chemoradiotherapy for esophageal adenocarcinoma. JAMA Surg. 2015;150:555–62. 25902198 10.1001/jamasurg.2014.3867

[26] Barron CC , Wang X , Elimova E . Neoadjuvant strategies for esophageal cancer: role of immunotherapy and positron emission tomography (PET)‐guided strategies. Thorac Surg Clin. 2023;33:197–208. 37045489 10.1016/j.thorsurg.2023.01.009

[27] Jayaprakasam VS , Paroder V , Schoder H . Variants and pitfalls in PET/CT imaging of gastrointestinal cancers. Semin Nucl Med. 2021;51:485–501. 33965198 10.1053/j.semnuclmed.2021.04.001 PMC8338802

[28] Long NM , Smith CS . Causes and imaging features of false positives and false negatives on F‐PET/CT in oncologic imaging. Insights Imaging. 2011;2:679–98. 22347986 10.1007/s13244-010-0062-3 PMC3259390

[29] Baker S , Fairchild A . Radiation‐induced esophagitis in lung cancer. Lung Cancer (Auckl). 2016;7:119–27. 28210168 10.2147/LCTT.S96443 PMC5310706

